# Deletion of Annexin A1 in Mice Upregulates the Expression of Its Receptor, Fpr2/3, and Reactivity to the AnxA1 Mimetic Peptide in Platelets

**DOI:** 10.3390/ijms24043424

**Published:** 2023-02-08

**Authors:** Olga Zharkova, Maryam F. Salamah, Maria V. Babak, Elanchezhian Rajan, Lina H. K. Lim, Frans Andrade, Cristiane D. Gil, Sonia M. Oliani, Leonardo A. Moraes, Sakthivel Vaiyapuri

**Affiliations:** 1 Immunology Program, Department of Physiology, Yong Loo Lin School of Medicine, National University of Singapore, Singapore 117593, Singapore; 2 School of Pharmacy, University of Reading, Reading RG6 6UB, UK; 3 Department of Chemistry, City University of Hong Kong, Hong Kong, China; 4 Department of Morphology and Genetics, Federal University of São Paulo (UNIFESP), São Paulo 04023-900, Brazil; 5 Department of Biology, Instituto de Biociências, Letras e Ciências Exatas (IBILCE), São Paulo State University (UNESP), São José do Rio Preto, São Paulo 15054-000, Brazil

**Keywords:** annexin A1, FPR2/ALX, thrombosis, inflammation, thromboinflammation, ANXA1_Ac2-26_

## Abstract

Annexin A1 (ANXA1) is an endogenous protein, which plays a central function in the modulation of inflammation. While the functions of ANXA1 and its exogenous peptidomimetics, *N*-Acetyl 2-26 ANXA1-derived peptide (ANXA1_Ac2-26_), in the modulation of immunological responses of neutrophils and monocytes have been investigated in detail, their effects on the modulation of platelet reactivity, haemostasis, thrombosis, and platelet-mediated inflammation remain largely unknown. Here, we demonstrate that the deletion of *Anxa1* in mice upregulates the expression of its receptor, formyl peptide receptor 2/3 (*Fpr2/3*, orthologue of human FPR2/ALX). As a result, the addition of ANXA1_Ac2-26_ to platelets exerts an activatory role in platelets, as characterised by its ability to increase the levels of fibrinogen binding and the exposure of P-selectin on the surface. Moreover, ANXA1_Ac2-26_ increased the development of platelet-leukocyte aggregates in whole blood. The experiments carried out using a pharmacological inhibitor (WRW4) for FPR2/ALX, and platelets isolated from *Fpr2/3*-deficient mice ascertained that the actions of ANXA1_Ac2-26_ are largely mediated through *Fpr2/3* in platelets. Together, this study demonstrates that in addition to its ability to modulate inflammatory responses via leukocytes, ANXA1 modulates platelet function, which may influence thrombosis, haemostasis, and platelet-mediated inflammation under various pathophysiological settings.

## 1. Introduction

Platelets, anucleated blood cells, play a primary role in the maintenance of haemostasis by preventing excessive bleeding upon vascular injury. Under various pathological settings, such as during the rupture of atherosclerotic plaque, the unnecessary activation of platelets results in the formation of blood clots (thrombosis) within the blood vessels [1]. The role of platelets in the regulation of haemostasis and thrombosis is well established but their immune-related functions have started receiving wider attention only in recent years. The platelet surface contains several immune/inflammatory receptors, e.g., toll-like receptors (TLRs), chemokine receptors, and formyl peptide receptors (FPRs), which engage with various soluble and/or fixed ligands of innate immune cells, thereby enabling platelet–leukocyte interactions [2]. These interactions play a critical role in the modulation of inflammatory responses under diverse pathophysiological settings [3]. Following acute damage or response to a pathological condition, inflammatory responses are induced, and these are later controlled by the resolution processes, which limit the migration of activated inflammatory cells and induce their apoptosis and clearance at the site of inflammation, thereby restoring homeostasis [4]. Initially, the resolution of acute inflammation is characterised by the recruitment of neutrophils, followed by the engagement of monocytes which subsequently differentiate into macrophages. During the resolution of acute inflammation, platelets are actively involved in different stages of neutrophil and other leukocyte recruitment [5]. Therefore, the formation of platelet–leukocyte aggregates may serve as a predictive biomarker of inflammation and thrombosis [6,7].

ANXA1, a key inflammatory molecule, plays a crucial role in the regulation of inflammation under various pathological conditions such as arthritis, cancer, and cardiovascular diseases. The molecular weight of ANXA1 is around 37 kDa with a C-terminal core region and an N-terminal functional region. The C-terminal region of this protein is composed of four homologous repeats each with around 70 amino acids. The N-terminal region contains 44 amino acids, of which the first 26 amino acids form two α-helices and the remaining region links this N-terminal region to the C-terminal core protein [8]. ANXA1 is normally expressed in different immune cells, e.g., monocytes, neutrophils, macrophages, and epithelial cells in airways [9]. The activation of these cells results in the secretion or externalisation of ANXA1 to the cell surface accompanied by the proteolytic cleavage of its N-terminal region to facilitate its binding to *N*-formyl peptide receptor 2/lipoxin A4 (FPR2/ALX) [8]. ANXA1 and its N-terminus mimetic peptide, *N*-Acetyl 2-26 (ANXA1_Ac2-26_) were demonstrated to exhibit various pro- and/or anti-inflammatory effects (based on the clinical settings) mediated by FPR2/ALX signalling axis [10]. However, their role in the modulation of platelet function, haemostasis, thrombosis, and platelet-mediated inflammation has not been fully established. Here, we investigated the impact of the deletion of the *Anxa1* gene (*Anxa1^−/−^*) in mouse platelets to demonstrate its significance in the regulation of platelet reactivity. Understanding the wider roles of ANXA1 and the significance of its receptor, FPR2/ALX in modulating platelet reactivity and platelet-mediated inflammation will lead to the development of improved therapeutic agents to control thromboinflammatory responses.

## 2. Results

### 2.1. Anxa1^−/−^ Mouse Platelets Exhibit Reduced GPIbα Level

**Figure 1:**
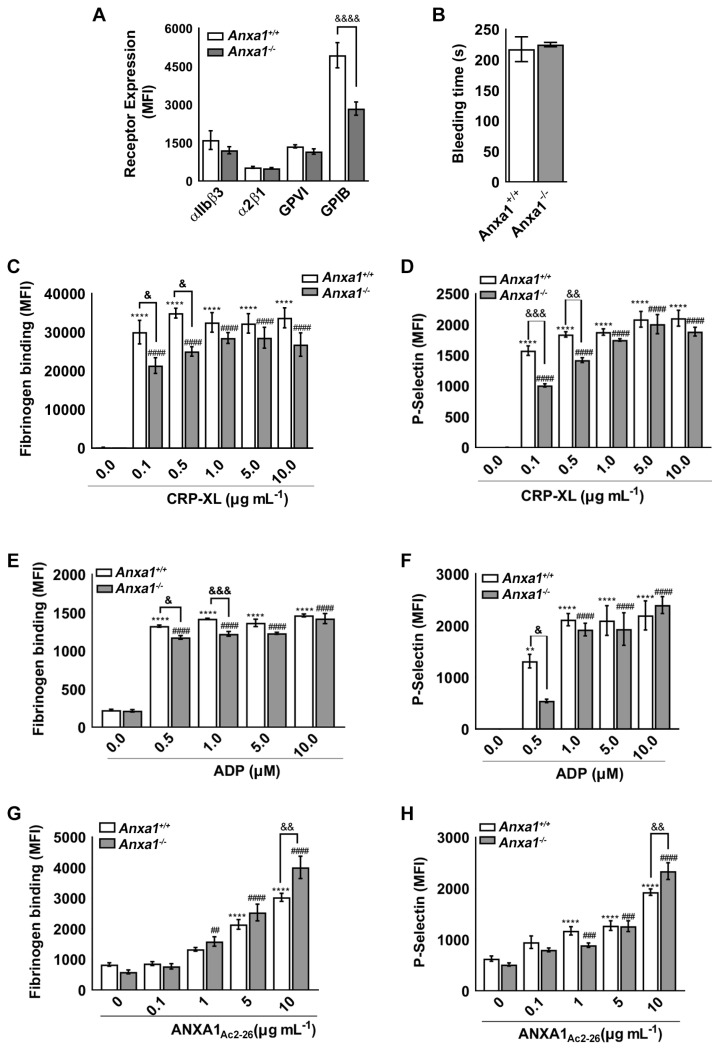
The characterisation and reactivity of platelets obtained from *Anxa1^−/−^* mice. (**A**) The expression of major receptors was analysed in platelets (whole blood) obtained from the control and *Anxa1^−/−^* mice using fluorescently labelled antibodies for specific receptors by flow cytometry. (**B**) Tail bleeding time in control and *Anxa1^−/−^* mice following the dissection of 3 mm tail tip. The amount of fibrinogen binding and exposure of P-selectin in platelets from the control and *Anxa1^−/−^* mice upon treatment with different concentrations of CRP-XL (**C**,**D**), ADP (**E**,**F**), and ANXA1_Ac2-26_ (**G**,**H**) were analysed using fluorescent-labelled anti-fibrinogen and P-selectin antibodies by flow cytometry. Data represent mean ± S.D. Statistical analysis was carried out using a two-way ANOVA test followed by Sidak’s multiple comparison tests (**A**) (*n* = 4 for each animal group), non-parametric Mann–Whitney test (**B**) (*n* = 7 for each animal group), two-way ANOVA for repeated measures with Tukey’s multiple comparisons test for (**C**–**H**) (*n* = 4 for each animal group). * *p* < 0.05 ** *p* < 0.01, *** *p* < 0.001 and **** *p* < 0.0001 (same applies to # and & symbols); ‘*’ and ‘#’ represent the comparison between the treated and untreated samples in the relevant mice group; ‘&’ represents the comparison between the control and *Anxa1^−/−^* mice in relevant data sets.

In this study, we used *Anxa1^−/−^* mice as one of the primary tools to investigate its functions in platelets. To determine if the deletion of the *Anxa1* gene in mice resulted in any defects in platelet characteristics, we measured the expression of key platelet surface receptors including integrin αIIbβ3, integrin α2β1, glycoprotein VI (GPVI), and GPIbα using whole blood withdrawn from the control and *Anxa1^−/−^* mice by flow cytometry (Figure 1A). While there was no difference detected in the levels of integrin αIIbβ3, integrin α2β1, and GPVI, a significant reduction in the level of GPIbα in platelets obtained from *Anxa1^−/−^* mice was observed in comparison to control mouse platelets. These data indicate that in mice, the deletion of *Anxa1* resulted in downregulation of the GPIbα level which may affect the function of platelets from these mice.

### 2.2. Haemostasis Is Unaffected in Anxa1^−/−^ Mice

Following the identification of reduced GPIbα levels in *Anxa1^−/−^* mouse platelets, we sought to determine if this change had been reflected in the maintenance of haemostasis in these mice. Therefore, a tail bleeding assay was carried out to determine if the deletion of *Anxa1* affected the haemostasis. The mean bleeding time observed for control (218 ± 54 s) and *Anxa1^−/−^* (225 ± 9 s) mice was not significantly different (Figure 1B). It was notable that all *Anxa1^−/−^* mice exhibited very similar bleeding times (deviation of 9 s) compared to those of the control group. This data suggests that the deletion of the *Anxa1* gene does not interfere with the maintenance of haemostasis, although there was a reduced level of GPIbα in platelets from *Anxa1^−/−^* mice.

### 2.3. ANXA1_Ac2-26_ Exhibits Increased Platelet Reactivity in Anxa1^−/−^ Mouse Platelets

To investigate whether the deletion of *Anxa1* can directly influence the platelet activation, the levels of fibrinogen binding (as a biomarker for inside-out signalling to integrin αIIbβ3) and exposure of P-selectin (as a biomarker for α-granule secretion) were measured using whole blood obtained from control and *Anxa1^−/−^* mice by flow cytometry. The whole blood from these mice was treated with commonly used platelet agonists such as cross-linked collagen-related peptide (CRP-XL, a specific agonist for GPVI receptor) or adenosine diphosphate (ADP, a selective agonist for P2Y receptors) prior to analysing the levels of fibrinogen binding and P-selectin. Upon treatment with lower concentrations of CRP-XL (0.1 and 0.5 μg mL^−1^), a significantly reduced level of fibrinogen binding (Figure 1C) and P-selectin (Figure 1D) was observed in platelets obtained from *Anxa1^−/−^* mice compared to the mice of the control group. However, at higher concentrations (1, 5 and 10 μg mL^−1^) of CRP-XL, there was no significant difference found in the level of platelet activity between control and *Anxa1^−/−^* platelets, although the reduction in platelet reactivity among *Anxa1^−/−^* platelets was notable. Similar data were attained when platelets were treated with different concentrations of ADP (Figure 1E,F).

The addition of exogenous ANXA1_Ac2-26_ activated platelets obtained from *Anxa1^−/−^* and control mice as shown by the levels of fibrinogen binding (Figure 1G) and P-selectin (Figure 1H) in a dose-dependent manner. However, the stimulation of platelets obtained from *Anxa1^−/−^* mice upon treatment with a high concentration (10 μM) of ANXA1_Ac2-26_ was significantly higher compared to the control mouse platelets.

These results demonstrate that there was a reduced level of platelet activation when low concentrations of CRP-XL and ADP were used, and this may be attributable to the reduced level of GPIbα. However, this effect did not exist when the concentrations of agonists were increased. This was also reflected in the lack of any noticeable effect in maintaining haemostasis in *Anxa1^−/−^* mice under physiological settings. Notably, ANXA1_Ac2-26_ was able to induce platelet activation and the level of activation was higher in platelets obtained from *Anxa1^−/−^* mice at a higher concentration of this peptide.

### 2.4. ANXA1_Ac2-26_ Induces Platelet Activation through FPR2/ALX

**Figure 2:**
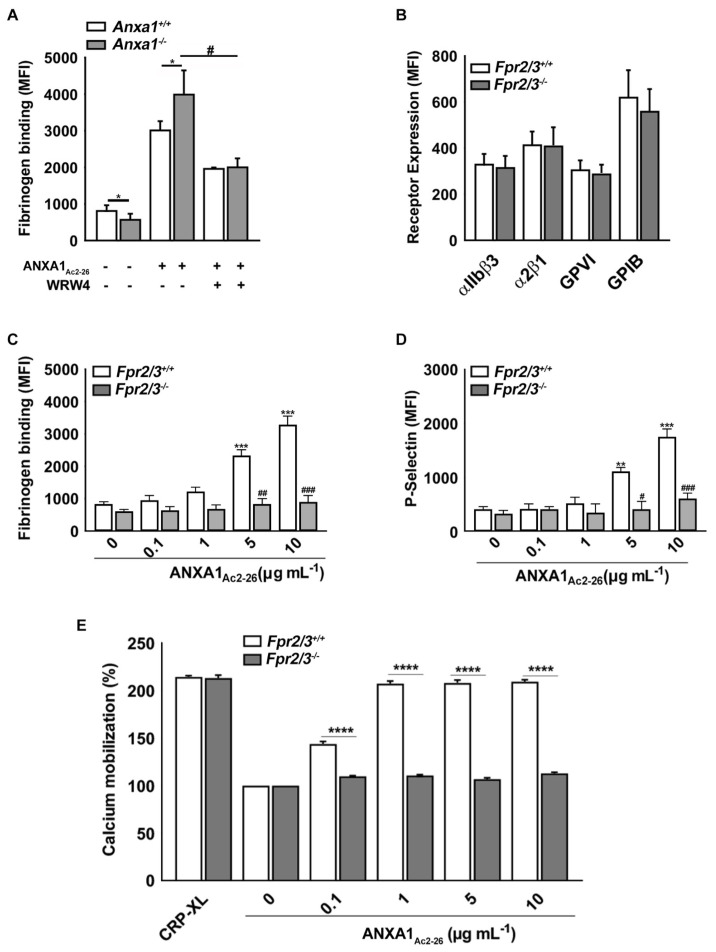
The role of FPR2/ALX in the modulation of ANXA1_Ac2-26_-mediated actions in platelets. (**A**) The amount of fibrinogen binding in platelets obtained from control and *Anxa1^−/−^* mice was quantified by flow cytometry upon treatment with ANXA1_Ac2-26_ (10 μg mL^−1^) in the presence or absence of WRW4 (5 μM). (**B**) The expression of major receptors in platelets from control and *Fpr2/3^−/−^* mice was analysed using fluorescently labelled antibodies against specific receptors by flow cytometry. The amounts of fibrinogen binding (**C**) and P-selectin (**D**) were analysed upon activation of platelets obtained from the control and *Fpr2/3^−/−^* mice upon treatment with diverse concentrations of ANXA1_Ac2-26_ by flow cytometry. (**E**) Calcium mobilisation in platelets obtained from the control and *Fpr2/3^−/−^* mice was analysed using Fluo-4 AM dye upon stimulation with 10 μg mL^−1^ of CPR-XL or various concentrations of ANXA1_Ac2-26_ by spectrofluorimetry. Data represent mean ± S.D. (*n* = 4). Statistical analysis was carried out by two-way ANOVA for repeated measures followed by Sidak’s multiple comparisons test for (**A**,**B**) and the two-way ANOVA test followed by Bonferroni’s multiple comparisons test for (**C**–**E**). * *p* < 0.05, ** *p* < 0.01, *** *p* < 0.001 and **** *p* < 0.0001 (same applies to # symbol); ‘*’ and ‘#’ represent the comparison between the treated and untreated samples in the relevant mice group in panels (**C**,**D**).

Following the determination of the impact of the deletion of *Anxa1* on the modulation of platelet function, we investigated whether these actions were mediated through *Fpr2/3*. Therefore, the whole blood samples withdrawn from the control and *Anxa1^−/−^* mice were treated with vehicle control or a selective FPR2/ALX-specific antagonist, WRW4 (5 μM; this concentration was selected based on our previous studies), for 5 min before stimulation with 10 μg mL^−1^ of ANXA1_Ac2-26_. The level of platelet activation was analysed by quantifying the amount of fibrinogen binding (as the level of increase in fibrinogen binding was more prominent (Figure 1G) upon stimulation with ANXA1_Ac2-26_ than in P-selectin exposure (Figure 1H)). While the amount of fibrinogen binding was increased in platelets from the control and *Anxa1^−/−^* mice upon treatment with ANXA1_Ac2-26_, this increase in fibrinogen binding was diminished by WRW4 (Figure 2A). To corroborate these findings, the platelets from *Fpr2/3^−/−^* (orthologue of human FPR2/ALX) mice were used. While the platelets obtained from *Fpr2/3^−/−^* mice did not show any defects in the expression of any major platelet surface receptors (Figure 2B), the effects of ANXA1_Ac2-26_ on the amount of fibrinogen binding (Figure 2C), P-selectin exposure (Figure 2D), and calcium mobilisation (Figure 2E) were largely reduced in these platelets. These data demonstrate that the actions of ANXA1_Ac2-26_ are largely mediated through FPR2/ALX in platelets.

### 2.5. Fpr2/3 Is Overexpressed in Anxa1^−/−^ Mouse Platelets

**Figure 3:**
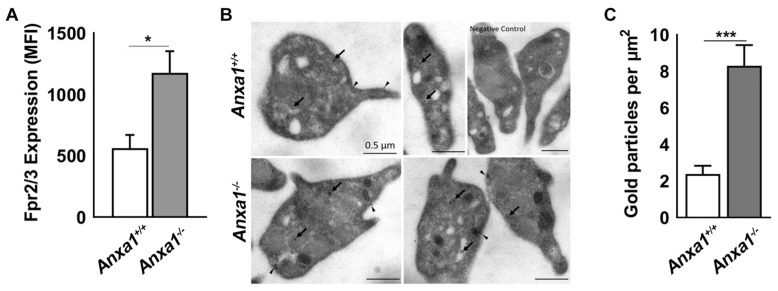
The expression of *Fpr2/3* in *Anxa1^−/−^* mouse platelets. (**A**) The expression levels of *Fpr2/3* were measured using selective antibodies by flow cytometry in platelets obtained from control and *Anxa1^−/−^* mice. (**B**) Ultrastructural localisation of *Fpr2/3* was analysed in platelet sections using selective antibodies against *Fpr2/3* and immunogold particles-conjugated secondary antibodies. The electron micrographs shown are representative of experiments performed with four separate mice in each group. The antibody binding to specific locations in the cytosol (arrows) and plasma membrane (arrowheads) is shown in comparison to control mouse platelets. The negative control images show the ultrastructure of platelet sections that were treated with secondary antibodies but in the absence of primary antibodies. (**C**) The density of *Fpr2/3* immunogold particles in platelets as quantified using electron micrographs. Data represent mean ± S.D. (*n* = 4 for **A**; multiple images from 4 mice in each group for **C**). The *p* values shown (* *p* < 0.05 and *** *p* < 0.001) are calculated by a paired Student *t*-test for (**A**) and a non-parametric Mann–Whitney test for (**C**).

Following the confirmation of the role of FPR2/ALX in mediating the functions of ANXA1_Ac2-26_ on platelets, we sought to investigate whether the level of *Fpr2/3* was altered in *Anxa1^−/−^* mice, as there was an increase in activation in platelets obtained from these mice when a higher concentration of ANXA1_Ac2-26_ was used (Figure 1G,H). Indeed, the level of *Fpr2/3* was significantly higher in platelets from *Anxa1^−/−^* mice compared to the controls, as demonstrated by flow cytometry (Figure 3A) and electron microscopy (Figure 3B,C). These data suggest that the deletion of *Anxa1* leads to increased expression of *Fpr2/3* in platelets from these mice.

### 2.6. ANXA1_Ac2-26_ Induces Platelet–Leukocyte Aggregates in the Whole Blood of Anxa1^−/−^ Mice

**Figure 4:**
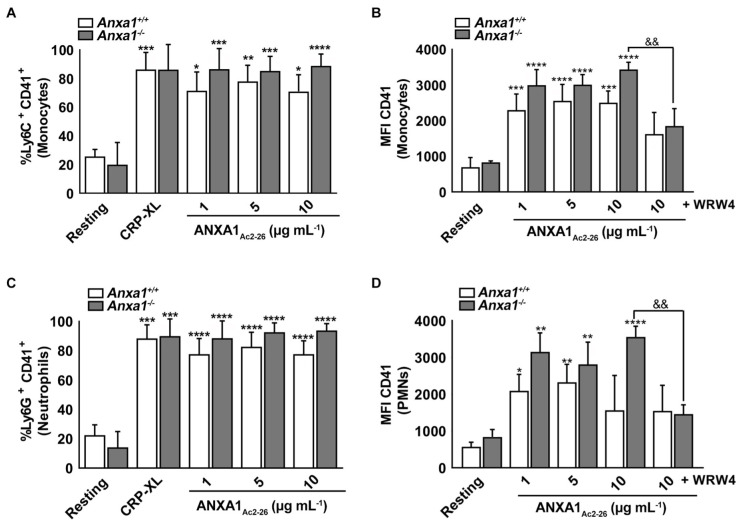
Effect of ANXA1_Ac2-26_ in the stimulation of platelet–leukocyte interactions. (**A**) The level (percentage) of platelet–monocyte aggregates within the total monocyte population in whole blood from control and *Anxa1^−/−^* mice upon treatment with CRP-XL (10 μg mL^−1^) or various concentrations of ANXA1_Ac2-26_. (**B**) The level of CD41 within platelet–monocyte aggregates in whole blood from control and *Anxa1^−/−^* mice upon incubation with various concentrations of ANXA1_Ac2-26_ in the presence or absence of WRW4 (5 μM). (**C**) The percentage of platelet–neutrophil aggregates within the total neutrophil population in whole blood obtained from the control and *Anxa1^−/−^* mice upon treatment with CRP-XL (10 μg mL^−1^) or different concentrations of ANXA1_Ac2-26_. (**D**) The level of CD41 in platelet–neutrophil aggregates in whole blood of the control and *Anxa1^−/−^* mice upon incubation with various concentrations of ANXA1_Ac2-26_ in the absence or presence of WRW4 (5 μM). Data represent mean ± S.D. (*n* = 4). Statistical analysis was carried out using two-way ANOVA for repeated measures followed by Sidak’s multiple comparisons test for (**A**,**C**) and Tukey’s multiple comparisons test for (**B**,**D**). * *p* < 0.05, ** *p* < 0.01, *** *p* < 0.001 and **** *p* < 0.0001 (same applies to & symbol); ‘*’ represents the comparison between the treated and untreated samples in relevant mice group; ‘&’ represents the comparison between the control and *Anxa1^−/−^* mice in relevant data sets.

Platelets are known to initiate/augment inflammatory responses through platelet–leukocyte aggregates [5]. To determine whether the deletion of *Anxa1* in mice affects the inflammatory responses, the development of platelet-leukocyte aggregates was examined in whole blood obtained from control and *Anxa1^−/−^* mice upon stimulation with ANXA1_Ac2-26_. Since platelets are generally characterised by CD41 (integrin αIIb), platelet–monocyte and platelet–neutrophil aggregates were analysed by measuring Ly6C^+^CD41^+^ and Ly6G^+^CD41^+^ populations, respectively, using a flow cytometer. The addition of different concentrations of ANXA1_Ac2-26_ enhanced platelet–monocyte (Figure 4A,B) and platelet–neutrophil (Figure 4C,D) interactions similar to CRP-XL (10 μg mL^−1^) in both control and *Anxa1^−/−^* mice. Notably, treatment with WRW4 significantly decreased the formation of platelet–monocyte (Figure 4B) and platelet–neutrophil (Figure 4D) aggregates. These data suggest that the deletion of *Anxa1* did not affect platelet interactions with leukocytes, especially neutrophils and monocytes, and ANXA1_Ac2-26_ induced these interactions via FPR2/ALX as expected. The effect of ANXA1_Ac2-26_ on both platelets and leukocytes is likely to regulate this process as they both express FPR2/ALX. Although it was not significant, the *Anxa1^−/−^* mouse platelets appeared to cause slightly more platelet–leukocyte interactions than those found in the control group in all these experiments and this could be attributed to the elevated levels of *Fpr2/3* in these mice.

## 3. Discussion

ANXA1 is an endogenous protein which has been demonstrated to play critical roles in the modulation of inflammation specifically during resolution to restore homeostasis [9]. It was previously used as an inhibitor for pro-inflammatory prostaglandins to investigate leukocyte aggregation and in numerous other settings [11]. Its anti-inflammatory and pro-resolving roles are well-established in different disease models including arthritis [12] and colitis [13]. However, various studies have reported the pro-inflammatory effects of ANXA1 [14,15]. ANXA1_Ac2-26_, a peptide derived from the N-terminus of ANXA1 has been shown to act similarly to its pharmacophore and possibly maintain the biochemical features of the full-length protein [16,17]. Both full-length protein and cleaved peptide were found in inflammatory exudates and other extracellular biological fluids during various diseases [8]. Similar to endogenous ANXA1, exogenous ANXA1_Ac2-26_ exhibited anti-inflammatory [9] and pro-inflammatory effects [18,19], which were mainly regulated by FPR family members, primarily FPR2/ALX [10].

While the modulatory effects of ANXA1 and ANXA1_Ac2-26_ during inflammation have been extensively studied in numerous disease models, their importance in platelet-mediated thromboinflammation is not yet fully understood. Recently, we reported the significance of FPRs and some of their ligands such as an antimicrobial peptide, LL37 [20,21], and a bacterial formyl peptide, fMLF [22], in the regulation of platelet function. Due to the critical roles of platelets at the interface between thrombosis and inflammation [3], the detailed characterisation of inflammatory receptors such as FPR2/ALX will pave the way to the development of novel therapeutic strategies to control exacerbated thromboinflammatory responses during pathological conditions. A recent study has demonstrated that the addition of full-length ANXA1 to platelets inhibited their activation and reduced their ability to aggregate, thereby preventing thrombosis under in vivo conditions [10]. Moreover, when ANXA1 was administered to mice before and after cerebral ischemia-reperfusion injury, the blood flow was significantly improved, indicating ANXA1-mediated an inhibitory effect on thrombosis as well as displayed its protective effects against recurrent post-stroke thrombotic events [10]. They also demonstrated that ANXA1 promoted the phagocytosis of human-activated platelets by neutrophils, thereby initiating the resolution of thromboinflammation [10].

Here, we demonstrated the impacts of the deletion of endogenous *Anxa1* in mice and its exogenous peptidomimetic ANXA1_Ac2-26_ on the modulation of platelet activation and platelet-mediated inflammation. The experiments performed using platelets obtained from *Anxa1^−/−^* mice demonstrated a slightly reduced activation of platelets compared to the control group when low concentrations of agonists were used. There was no significant reduction in platelet reactivity when high concentrations of agonists were used, although the activation being clearly reduced. This may relate to the reduced level of GPIbα expression observed in *Anxa1^−/−^* mouse platelets, although it did not affect the overall haemostasis in these mice. There were no other major differences observed in platelet reactivity, haemostasis, or expression of specific platelet surface receptors in *Anxa1^−/−^* mice. Similar observations were noted in a previous study that analysed the significance of ANXA1 in the regulation of post-thrombotic events following a stroke [10]. These results suggest that ANXA1 may only act at the site of injury while leaving the physiological haemostasis intact. Indeed, ANXA1 will only be released from activated immune cells at the site of inflammation. Contrary to endogenous full-length ANXA1 which appeared to inhibit platelet activation, ANXA1_Ac2-26_ caused the dose-dependent activation of platelets, which was comparable in both control and *Anxa1^−/−^* mice at 1–5 μg mL^−1^ concentrations, although the platelet activation in *Anxa1^−/−^* mouse platelets was higher at 10 μg mL^−1^ concentration of this peptide. This increase in platelet activation may be attributed to the elevated expression of *Fpr2/3* in *Anxa1^−/−^* mouse platelets as a compensatory mechanism implicated with the deficiency of *Anxa1* in these mice, aiming to overcome the effects due to its deletion [23,24]. However, the differences in the actions of full-length ANXA1 and ANXA1_Ac2-26_ should be clarified in future studies. These differences may be due to the nature of experiments performed, as this present study only demonstrates the effects of ANXA1_Ac2-26_ under ex vivo settings, whereas the previous study reported the impact of full-length ANXA1 in a disease model under in vivo circumstances. Moreover, the efficacy and binding of full-length ANXA1protein and ANXA1_Ac2-26_ peptide might be different. The synergistic effects of other factors, such as circulating inflammatory molecules influencing the roles of ANXA1, cannot be ruled under in vivo settings.

Since FPR2/ALX signalling axis is known to play a crucial role in the regulation of ANXA1- and ANXA1_Ac2-26_-mediated functions, we investigated the significance of ANXA1_Ac2-26_ using platelets from *Fpr2/3^−/−^* mice and a selective pharmacological antagonist for FPR2/ALX, WRW4. This inhibitor contains six amino acids (WRWWWW) and it has been shown to selectively inhibit FPR2/ALX and affect downstream functions such as calcium mobilization in neutrophils. The activation of platelets by ANXA1_Ac2-26_ was largely reduced by WRW4. We also previously demonstrated that WRW4 was able to affect positive feedback activation of platelets when stimulated with commonly used platelet agonists, e.g., CRP-XL, ADP, and AY-NH_2_. As expected, the activation of platelets by ANXA1_Ac2-26_ was significantly lower in *Fpr2/3^−/−^* mouse platelets compared to the controls. Additionally, the ability of ANXA1_Ac2-26_ to induce calcium mobilisation, as this is essential for platelet activation [25] was analysed in platelets from *Fpr2/3^−/−^* mice. Indeed, ANXA1_Ac2-26_-induced calcium mobilisation in *Fpr2/3^−/−^* mouse platelets was largely reduced, further supporting the notion that ANXA1_Ac2-26_ mediates its functions via FPR2/ALX in platelets.

Subsequently, the role of ANXA1 and ANXA1_Ac2-26_ in platelet-mediated inflammation was analysed. Many functions of platelets as immune cells involve an interplay with leukocytes via direct interactions or release of several inflammatory mediators [26]. ANXA1_Ac2-26_ stimulated platelet–monocyte and platelet–neutrophil interactions, which were abrogated in the presence of WRW4, suggesting that the immune functions of ANXA1 also depend on FPR2/ALX. *Anxa1^−/−^* mouse platelets showed slightly more interactions with leukocytes, although it was not significant, and this may relate to the upregulation of *Fpr2/3* expression in these mice. A protective role for ANXA1 in various pathological conditions that are implicated with augmented platelet activation has been reported previously, e.g., atherosclerosis [27,28], myocardial infarctions [29], and strokes [10]. The diverse effects of ANXA1 and ANXA1_Ac2-26_ observed could be attributed to the ability of ANXA1_Ac2-26_ to heterodimerise FPR2/ALX with FPR1, leading to the activation of pro-apoptotic signalling pathways [30]. Furthermore, ANXA1_Ac2-26_ is known to activate all FPR family members, although it mainly exerts its effects through FPR2/ALX [31,32], while ANXA1 binds only to FPR2/ALX [33,34]. Notably, pro-inflammatory responses upon ligand binding to FPR receptors are mainly elicited via FPR1, while the majority of anti-inflammatory and pro-resolving functions are regulated through FPR2/ALX [35]. The activation of *Fpr2/3* in neutrophils by aspirin-triggered lipoxin A4 has affected the formation of neutrophil and platelet aggregates during cerebral ischemia/reperfusion injury [35]. Nonetheless, some ligands can activate pro-inflammatory actions through FPR2/ALX, such as the endogenous antimicrobial peptide, LL37 [22]. Although it is well established that ANXA1 exerts its effects through FPR2/ALX, further research is required to determine the role of other FPRs in the regulation of ANXA1 and ANXA1_Ac2-26_-mediated thromboinflammatory responses specifically via platelets. Moreover, the actions of ANXA1_Ac2-26_ observed in mouse platelets in this study should also be corroborated using human platelets in future research, as there may be some differences in the level and nature of its modulatory effects.

Deciphering the actions of the FPR2/ALX in the regulation of ANXA1-mediated platelet function offers new avenues to develop better treatment strategies for the management of thromboinflammatory responses, particularly those associated with bleeding or thrombotic events using this as a powerful therapeutic target. Furthermore, it could provide novel insights into the mechanisms underlying platelet-associated complications in diverse inflammatory diseases including sepsis, atherosclerosis, and notably, COVID-19 in which platelets were demonstrated to have significant roles via various mechanisms [36]. Since various pro-resolving mechanisms are mediated through FPR2/ALX, it could be targeted to suppress exacerbated inflammation without affecting the host’s defence, thereby minimising unwarranted side effects while achieving effective therapeutic strategies [37].

In addition to our previous studies, here we demonstrate the biological significance of ANXA1 and its N-terminal peptide ANXA1_Ac2-26_ in the modulation of platelet reactivity and platelet-mediated inflammatory responses. Notably, this study further emphasises the critical roles of FPR2/ALX in the modulation of platelet function and the significance of platelets in the modulation of thromboinflammation. In addition to the impact of LL37, the significance of ANXA1 and its derivative in the modulation of thrombosis, haemostasis, and platelet-mediated inflammation via FPR2/ALX renders them useful targets to control the exacerbation of thrombotic complications and inflammatory responses in numerous pathological settings where unwarranted platelet activation is a major concern. Hence, the platelet FPR2/ALX may act as a robust therapeutic target to control unwarranted thromboinflammatory responses under diverse clinical scenarios.

## 4. Materials and Methods

All the experiments in this study were performed in accordance with the relevant guidelines and regulations as set out by appropriate authorities. In addition, this study was carried out in compliance with the ARRIVE guidelines.

### 4.1. Animals

*Anxa1^−/−^* mice on the C57BL/6 background were obtained from Professor Roderick Flower at Queen Mary University of London (UK). These mice were developed by replacing a portion of exon 2 and full of exons 3 and 4 of the *Anxa1* gene with a reporter cassette resulting in the absence of endogenous protein in homozygous mutant mice. Age-matched control mice (8–12 weeks) were used in all the experiments. These were bred and maintained in-house at the Laboratory Animal Centre (Singapore) where *Anxa1^−/−^* mice were also maintained. All animal works using *Anxa1^−/−^* mice and controls performed were approved by the Institutional Animal Care and Use Committee and the National Advisory Committee for Laboratory Animals Research (NACLAR) at the National University of Singapore.

*Fpr2/3^−/−^* on a C57BL/6 background were received from Professor Mauro Perretti at William Harvey Research Institute, London (UK). The control mice that were used in the UK were originally obtained from Envigo, UK, and then they were bred and maintained in-house at the University of Reading, where *Fpr2/3^−/−^* mice were also housed. The procedures (for calcium mobilisation and flow cytometry assays) performed at the University of Reading were approved by the British Home Office, UK.

The fixed/processed platelet samples were sent to the Federal University of Sao Paulo, Brazil to perform electron microscopy experiments.

### 4.2. Preparation of Mouse Platelets

The mouse blood collection and preparation of platelets were performed as described previously [20,38,39,40]. The mice were sacrificed using CO_2_ and the blood was directly obtained via cardiac puncture into a syringe with 3.2% (*w*/*v*) sodium citrate at a 1:9 ratio. Then, the blood was centrifuged at room temperature at 203× *g* for 8 min and the platelet-rich plasma (PRP) was collected. 500 µL of modified Tyrode’s-HEPES buffer was used to suspend the remaining blood prior to centrifuging again at 203× *g* for 5 min. The resulting PRP was centrifuged at 1028× *g* for 5 min. The resulting platelet pellet was then resuspended in a modified Tyrode’s-HEPES buffer at a concentration of 2 × 10^8^ cells/mL. Notably, endotoxin-free water was used in all the experiments to avoid any unwarranted inflammatory responses from pathogenic contaminants, e.g., LPS.

### 4.3. Tail Bleeding Assay in Mice

The tail bleeding experiment was carried out as described previously [38,41,42]. In brief, the control or *Anxa1^−/−^* mice were anaesthetised using xylazine (5 mg/kg) and ketamine (80 mg/kg) administered through the intraperitoneal route for 20 min before the experiment. The mice were then placed on a heated mat at 37 °C. The tail tip (3 mm) was sliced using a scalpel blade and then the tail tip was placed in sterile saline prewarmed at 37 °C. The bleeding time was monitored for up to 20 min at which point the assay was stopped.

### 4.4. Intracellular Calcium Mobilisation Assay

The calcium mobilisation assay was carried out as reported previously [20,43,44]. PRP (2 mL) derived from the control and *Fpr2/3^−/−^* mice was mixed with 1 µM Fluo-4 AM dye (Life technologies, Paisley, UK) and incubated for 20 min at 30 °C in the dark. The PRP was later centrifuged at 1413× *g* for 10 min at 20 °C. The resulting platelet pellet was suspended in 500 µL Tyrode’s-HEPES buffer and maintained at 30 °C in the dark. The platelets were stimulated with various concentrations of ANXA1_Ac2-26_ (0.1–10 μg mL^−1^; prepared in endotoxin-free water) (Tocris, Bristol, UK) or 1 μg mL^−1^ of CRP-XL and the amount of fluorescence intensity was measured by a FluoStar Optima spectrofluorimeter (BMG Labtech, Ortenberg, Germany) at 37 °C for 180 s using an excitation wavelength of 485 nm and emission wavelength at 510 nm. Data were analysed by quantifying the level of calcium released at 90 s.

### 4.5. Electron Microscopy Analysis

The mouse isolated platelets were fixed in 4% (*w*/*v*) paraformaldehyde and 0.5% (*v*/*v*) glutaraldehyde solution (1:1) prepared in sodium cacodylate buffer 0.1 M (pH 7.4) for 24 h at 4 °C. The platelets were then dehydrated through a methanol series and then embedded in LRGold resin (London Resin, London, UK). To detect the localisation of the *Fpr2/3*, ultrathin sections (70 nm) were treated with the rabbit polyclonal anti-FPR2/ALX antibodies (1:100; Santa Cruz Biotechnology, Dalas, TX, USA) and goat anti-rabbit IgG (1:50 in PBS containing 1% egg albumin) conjugated with colloidal gold (15 nm) (British Biocell, Cardiff, UK). These sections were treated with uranyl acetate and lead citrate prior to examination using a ZEISS EM900 electron microscope (Carl Zeiss, Berlin, Germany). Photographed (randomly) sections of cells were used for immunocytochemical analysis. The area of the cell compartment was investigated with AxioVision software (version 4.8). The density of immunogold particles (gold particles per μm^2^) was quantified and expressed for each compartment. Data were reported as mean ± SEM of 25–30 electron micrographs analysed per group.

### 4.6. Flow Cytometry-Based Assays

The amounts of fibrinogen binding and P-selectin were quantified according to the previously published protocols [38,43,45]. Five microlitres of whole blood were incubated with increasing concentrations of CRP-XL, ADP, and ANXA1_Ac2-26_ (R & D Systems, Abingdon, UK) for 5 min or 100 nm PMA for 4 h at room temperature. 1 µL of FITC-conjugated fibrinogen antibodies and 1 µL of PE-Cy5-conjugated anti-CD62P (P-selectin) antibodies were used to detect the level of fibrinogen binding and exposure of P-selectin, respectively. To measure platelet–leukocyte aggregates, the whole blood (following red cell lysis) was stained for 20 min at room temperature with a cocktail of antibodies including FITC-labelled anti-mouse CD41 (Clone MWReg30), Alexa Fluor^®^-labelled 700 anti-mouse Ly6G (clone 1A8), and PE-labelled anti-mouse Ly6C (clone HK1.4) antibodies (all from Biolegend, San Diego, CA, USA). Following stimulation, the cells were washed (three times) prior to analysis by flow cytometry. The multiparameter acquisition was carried out using the LRS Fortessa flow cytometer (BD Biosciences, San Jose, CA, UK). The mean fluorescence intensity was estimated using the FlowJo tool (Tree Star, Ashland, OR, USA) to quantify the amounts of fibrinogen binding, P-selectin expression on the platelet surface, and platelet–leukocyte interactions. Negative controls were set using isotype-matched control antibodies. Similarly, for the analysis of expression of ANXA1 and FPR2/ALX, samples were incubated with 1 µL of anti-ANXA1 or anti-FPR2/ALX (5 µg mL^−1^) antibodies followed by 2 µL of Cy5-conjugated secondary antibodies. Following 20 min of incubation at room temperature, samples were fixed in 0.2% (*v*/*v*) formyl saline and analysed using flow cytometry. In experiments requiring the use of selective FPR2/ALX inhibitor, platelets were pretreated with 5 μM WRW4 (prepared in endotoxin-free water) (Tocris, UK) for 5 min and then treated with an appropriate concentration of ANXA1_Ac2-26_ for 5 min prior to analysis.

### 4.7. Statistical Analysis

The data obtained from various experiments are presented as mean ± SEM. Results were confirmed to follow a normal distribution as determined using the D’Agostino and Pearson normality test, the Shapiro–Wilk normality test or the Kolmogorov–Smirnov test of normality with the corrected Lilliefors’ test for normality by Dallal–Wilkinson. Data which failed the normality assumption were analysed using the non-parametric Mann–Whitney test. Statistical analysis was performed by a two-way ANOVA test followed by Sidak’s or Tukey’s multiple comparisons test unless otherwise specified. All the statistical analyses were carried out using Graphpad Prism 8.4.1 software (GraphPad Software Inc., Boston, MA, USA).

## Data Availability

All data are provided in this article.
